# Clinician-reported barriers to using exposure with response prevention in the treatment of paediatric obsessive-compulsive disorder

**DOI:** 10.1016/j.jocrd.2019.100498

**Published:** 2020-01

**Authors:** Julia Keleher, Amita Jassi, Georgina Krebs

**Affiliations:** a King's College London, Department of Psychosis Studies, Institute of Psychiatry, De Crespigny Park, London, UK; b National and Specialist OCD, BDD and Related Disorders Clinic for Young People, South London and Maudsley NHS Foundation Trust, London, UK; c King's College London, MRC Social, Genetic and Developmental Psychiatry Centre, Institute of Psychiatry, De Crespigny Park, London, UK

**Keywords:** Obsessive-compulsive disorder, Paediatric, Cognitive behaviour therapy, Barriers, Dissemination, Exposure therapy, Exposure with repsonse prevention

## Abstract

Exposure techniques are underutilised in the treatment of anxiety disorders in routine practice, but little is known about the use of exposure with response prevention (ERP) for OCD, particularly in youth. The current study aimed to examine the utilisation of ERP for paediatric OCD via an anonymous online survey completed by clinicians (*N* = 107). Specifically, we explored the association of clinician characteristics and OCD symptom subtypes with ERP use, as well as clinician-reported barriers to ERP implementation. The majority of clinicians reported commonly using ERP when treating youth with OCD, and rates of ERP use were highest among clinical psychologists. Clinician-held negative beliefs about exposure were significantly associated with lower ERP use. Additionally, clinicians reported being less likely to use ERP to treat hoarding symptoms and taboo obsessions, compared to other OCD symptom subtypes. The most commonly endorsed barriers to successful ERP implementation were aspects of the phenomenology of OCD (e.g. covert compulsions, frequently changing rituals) as opposed to general barriers (e.g. insufficient time during sessions). Overall, our findings suggest that OCD presents unique challenges for clinicians delivering exposure-based therapy. Training should address these OCD-specific obstacles in order to promote dissemination of ERP for youth with OCD.

## Highlights

• Three-quarters of clinicians frequently used ERP to treat paediatric OCD.
• Clinician-held negative beliefs about exposure were associated with lower ERP use.
• ERP was used less frequently to treat taboo obsessions and hoarding symptoms.
• The most common barriers to successful ERP implementation were OCD-specific.
• These included covert rituals, constantly changing rituals, and no clear obsession.

## 1 Introduction

Obsessive-compulsive disorder (OCD) is a common condition, affecting approximately 2% of young people (Heyman et al., 2001). The disorder is highly debilitating and without treatment symptoms typically persist into adulthood (Stewart et al., 2004). Cognitive behaviour therapy (CBT) has been well-established as an efficacious treatment for paediatric OCD (Watson & Rees, 2008) and is the recommended first-line treatment (NICE, 2005). Importantly, clinical guidelines specify CBT for OCD should focus on exposure with response prevention (ERP), as it is widely considered to be the main active element of this treatment (NICE, 2005). ERP consists of confronting obsessional fears (exposure) whilst simultaneously resisting carrying out a compulsion to neutralise anxiety or a feared outcome (response prevention). ERP tasks are set up in graded way, guided by a hierarchy, and are carried out in sessions with the therapist and in between sessions as homework (e.g. Turner, Krebs, & Volz, 2019).

Despite robust empirical support for ERP in the treatment of OCD, little is known about its application in routine clinical practice. There is considerable evidence indicating an underutilisation of exposure-based treatments more widely for anxiety disorders in both adults (Becker, Zayfert, & Anderson, 2004; Hipol & Deacon, 2012) and young people (Reid et al., 2017; Whiteside, Deacon, Benito, & Stewart, 2016). Previous studies have identified a range of factors associated with the use of exposure techniques including: a) clinician-related factors, such as professional background, therapeutic orientation, specialist training, therapist anxiety, and therapists’ beliefs about exposure (Becker et al., 2004; Harned, Dimeff, Woodcock, & Contreras, 2013; Farrell, Deacon, Kemp, Dixon, & Sy, 2013; Reid et al., 2017; Whiteside et al., 2016); b) organisational factors, including session length, lack of supervision and support personnel, work place policies, and lack of resources for exposure work (Moritz et al., 2019; Reid et al., 2017); and c) patient and family factors, such as psychiatric comorbidity, symptom severity, lack of motivation and concern about parental reaction (Becker et al., 2004; Moritz et al., 2019; Reid et al., 2017; Van Minnen, Hendriks, & Olff, 2010).

To our knowledge, only three studies have investigated therapist-reported use of ERP for OCD specifically (Jacobson, Newman, & Goldfried, 2016; Moritz et al., 2019; Valderhaug, Gö;testam, & Larsson, 2004), only one of which focussed on paediatric OCD (Valderhaug, Götestam, & Larsson, 2004). The estimated rates of ERP utilisation varied substantially across studies, ranging from 33% (Valderhaug et al., 2004) to 96% (Jacobson et al., 2016). Possible explanations for these discrepancies include differences in sample characteristics (e.g. level of expertise) as well as the time lag between studies. Nevertheless, there is a clear need to further clarify rates of ERP use in the treatment of OCD, particularly in youth.

Of note, the only previous study to investigate barriers to ERP use in OCD focused on generic obstacles (e.g. organizational issues) (Moritz et al., 2019). No previous study has examined whether specific characteristics of OCD may in themselves interfere with ERP utilisation. OCD is a highly heterogeneous condition (Mataix-Cols, Nakatani, Micali, & Heyman, 2008) and it is possible that different presentations of OCD are associated with unique challenges for clinicians when implementing ERP. For example, a therapist may have ethical concerns about exposure tasks for a young person suffering from taboo obsessions such as inappropriate sexual thoughts. Similarly, clinicians often view mental rituals as being particularly challenging to tackle with ERP (Gillihan, Williams, Malcoun, Yadi, & Foa, 2012) and may therefore adopt non-exposure strategies when treating this presentation. In addition to the specific symptoms, other aspects of the phenomenology of OCD may impact on ERP use. Young people with OCD often struggle to articulate their obsessional fear, which may deter therapists from using exposure techniques (Jacobson et al., 2016). Even when symptoms are identified, they can appear to shift and change over time (Valderhaug et al., 2004), making it hard for clinicians to plan a program of ERP.

The present study aimed to contribute to the small literature on use of ERP for OCD in routine clinical practice. Specifically, our first aim was to assess the frequency of clinician-reported ERP use for the treatment of OCD in youth in the United Kingdom (UK) as this is poorly understood currently. We hypothesised that a substantial proportion of clinicians would not use ERP to treat OCD. Our second aim was to establish clinician-related factors associated with ERP use (e.g. professional background). Based on previous research on exposure therapy for anxiety disorders, we hypothesised that clinicians who reported using ERP would have fewer negative beliefs about exposure than those who did not. Our third aim was to explore the utilisation of ERP for different OCD symptom dimensions. Based on clinical experience, we hypothesised that clinicians would be less likely to attempt ERP when treating taboo obsessions (e.g. sexual obsessions), compared to other symptom domains. Our final aim was to explore clinician-reported obstacles to the successful implementation of ERP in the treatment of paediatric OCD. We hypothesised that OCD-specific factors (e.g. mental rituals, constantly changing compulsions) would be reported as common barriers to the successful implementation of ERP.

## 2 Methods

### 2.1 Participants

The sample (*N* = 107) comprised both private (50%, *n* = 53) and National Health Service (NHS) mental health service employees (50%, *n* = 54). The characteristics of participating clinicians are shown in detail in Table S1 of the supplementary materials. In summary, 55% of the sample were qualified clinical psychologists (*n* = 59), with the remainder including trainee clinical psychologists (5%, *n* = 5), psychiatric nurses (13%, *n* = 14), psychiatrists (8%, *n* = 9) and social workers (5%, *n* = 5). The majority of clinicians (85%, *n* = 91) reported CBT as their main therapeutic orientation. Participants were of mixed experience levels, ranging from less than a year (22%, *n* = 23) to at least 17 years of experience (13%, *n* = 14), with a median of 5–8 years. The number of paediatric OCD cases that clinicians had treated with therapy ranged from 1 to 3 (38%, *n* = 41) to over 15 cases (24%, *n* = 26), with a median of 4–6 cases. The amount of supervision received ranged from none (3%) to over 3 h per month (27%), with a median of 2 h per month.

### 2.2 Procedure

Ethical approval for this study was granted by the King's College London (KCL) Psychiatry, Nursing & Midwifery Research Ethics Subcommittee (RESCM-17/18–3998). Health Research Authority (HRA) approval was sought to recruit NHS employees within South London and Maudsley NHS Foundation Trust (IRAS project ID: 222490). The sample was recruited via an email containing brief information about the study and a link to the online survey. The email was sent to gatekeepers (e.g. service managers or clinical directors) of private and NHS child and adolescent mental health services, who were asked to circulate the email to employees. Individuals who accessed the online survey were taken to a webpage containing information about the study and were given the option to consent to participate. Data were only saved for participants who chose to submit their responses at the end of the survey. Respondents were informed that the survey was about ‘therapy available for young people with OCD’. They were not told that the study was specifically focussed on ERP, in order to minimise selection and response bias. Respondents could be of any professional background but were required to have self-reported experience of providing therapy to at least one child or adolescent with OCD, assessed by a screening question at the start of the survey. In the UK, although clinical psychologists are one of the main providers of psychological therapies, these interventions are also commonly delivered by a range of other mental health professionals, especially in child and adolescent mental health services (Scott, 2018).

### 2.3 Measures

*Therapeutic Techniques*. Participants were asked how frequently they used 21 techniques from a range of therapeutic modalities to treat paediatric OCD. Items were scored on a 5-point scale ranging from 0 (‘never’) to 4 (‘always’). The list of techniques was adapted from Whiteside et al. (2016) and included exposure items (e.g. therapist-assisted exposure) as well as a range of other techniques (e.g. thought distraction, motivational interviewing etc.).

In order to assess the frequency with which clinicians utilised ERP specifically, participants were presented with a definition of ERP, before being asked: ‘How often do you use this therapeutic approach in your treatment for children and/or adolescents diagnosed with OCD?‘. This was scored on same 5-point scale as described above.

*The Therapist Beliefs about Exposure Scale (TBES;* Deacon, Farrell, Kemp, Dixon, & Sy, 2013*).* The TBES is a 21-item measure that assesses a range of negative beliefs that therapists might hold about exposure therapy (e.g. ‘Most clients have difficulty tolerating the distress exposure therapy evokes’). Items are scored on a 5-point scale ranging from 0 (‘disagree strongly’) to 4 (‘agree strongly’), yielding a total score of 0–84. The TBES has a single-factor structure and strong psychometric properties including high levels of internal consistency (α = 0.90–96) and test-retest reliability over a six-month period (r = 0.89) (Deacon et al., 2013). Cronbach's alpha for the present sample was .89.

*ERP Use for Different OCD Symptom Dimensions*. Participants were asked how likely, ranging from 0 (‘never’) to 4 (‘always’), they would be to use ERP to treat six different OCD symptom domains in children and/or adolescents, based on a previous factor analytic study of OCD symptom dimensions in youth (Mataix-Cols et al., 2008), with some adaptations based on our clinical experience: contamination fears/cleaning rituals, taboo obsessions (e.g. sexual obsessions), aggressive obsessions, symmetry/ordering compulsions, hoarding, and mental rituals.

*Barriers to ERP Use*. Participants were asked about nine potential barriers to implementing ERP when treating paediatric OCD (see Table S2). All items were derived from previous literature (e.g. Becker et al., 2004; Freeman et al., 2012; Reid et al., 2017; Valderhaug et al., 2004) and were selected based on our extensive clinical experience of delivering ERP to youth with OCD and training other mental health professions on this treatment approach. Responses were rated on a 5-point scale ranging from 0 (‘never’) to 4 (‘always’). For example, participants were presented with the statement, ‘I find it difficult to implement response prevention strategies because the young person's compulsions are constantly changing’. Participants who did not report experience of using ERP were asked to respond to these items hypothetically.

### 2.4 Analytic approach

Data were analysed using Stata version 14.2. Descriptive statistics were used to examine use of different therapeutic techniques, ERP use for different symptom domains and barriers to ERP use. Chi-squared tests and an independent samples *t*-test were used to examine factors associated with ERP use. A logistic regression was carried out to determine which factors were unique predictors of frequent ERP use.

## 3 Results

### 3.1 Utilisation of different therapeutic techniques

Table 1 illustrates the reported use of different therapeutic techniques. The techniques that were most commonly endorsed by clinicians as being used ‘often’ or ‘always’ were: exposure as homework (endorsed by 78% of sample); graded reduction of rituals (endorsed by 73%); therapist assisted in vivo exposure (endorsed by 69%); and identifying emotions (endorsed by 68%).

**Table 1:** Clinicians’ self-reported utilisation of specific therapeutic techniques to treat paediatric OCD.

Technique	Mean *(SD)*	Never Used (%)	Rarely Used (%)	Moderately Used (%)	Often Used (%)	Always Used (%)
**In vivo exposure as homework**	**4.23 (1.19)**	**5.6**	**5.6**	**10.3**	**16.8**	61.7*
**Graded reduction of rituals**	**4.11 (1.09)**	**3.7**	**4.7**	**17.8**	**24.3**	49.5*
Identifying emotions	4.05 (0.97)	0.9	3.7	27.1	26.2	42.1
**Therapist assisted in vivo exposure (i.e. direct exposure to fears in real life)**	**3.99 (1.15)**	**1.9**	**13.1**	**15.9**	**22.4**	46.7*
Cognitive restructuring	3.55 (1.07)	1.9	15.9	29.9	29.9	22.4
**Delaying rituals**	**3.38 (1.05)**	**2.8**	**16.8**	**37.4**	**25.2**	**17.8**
**Therapist assisted imaginal exposure (i.e. imagining the feared situation)**	**3.15 (1.18)**	**11.2**	**16.8**	**29.9**	**29.9**	**12.1**
Problem solving	3.04 (1.10)	9.3	23.4	28.0	32.7	6.5
Breathing/Relaxation techniques	3.0 (1.41)	18.7	23.4	16.8	21.5	19.6
Thought distraction techniques	2.83 (1.42)	24.3	20.6	18.7	20.6	15.9
Motivational interviewing	2.80 (1.15)	11.2	34.6	25.2	20.6	8.4
Family therapy techniques	2.75 (1.15)	15.0	29.0	29.0	18.7	7.5
**Therapist assisted introceptive exposure (i.e. exposure to bodily sensations**	**2.70 (1.28)**	**20.6**	**27.1**	**25.2**	**15.9**	**11.2**
Mindfulness techniques	2.67 (1.24)	19.6	31.8	17.8	23.4	7.5
Positive imagery	2.38 (1.23)	29.0	32.7	15.0	17.8	5.6
**Imaginal exposure as homework**	**2.36 (1.21)**	**29.9**	**29.9**	**18.7**	**16.8**	**4.7**
**Introceptive exposure as homework**	**2.25 (1.33)**	**41.1**	**22.4**	**14.0**	**15.0**	**7.5**
Art therapy techniques	1.37 (0.75)	75.7	14.0	7.5	2.8	–
Psychodynamic/Analytical techniques	1.35 (0.73)	77.6	13.1	6.5	2.8	–
Play therapy techniques	1.34 (0.67)	75.7	17.8	3.7	2.8	–
Eye Movement Desensitisation and Reprocessing (EMDR)	1.08 (0.39)	94.4	3.7	0.9	0.9	–

*Note:* Text in **bold** indicates ERP techniques - * indicates 1st, 2nd or 3rd most commonly endorsed as used ‘often’ or ‘always’.

### 3.2 Overall use of ERP as an approach

When presented with a definition of ERP and asked directly about their use of the approach, 75% (*n* = 80) of the sample reported using this technique ‘often’ or ‘always’ when treating children and/or adolescents with OCD. Only 5% (*n* = 5) of the sample reported never using ERP as a treatment for paediatric OCD.

### 3.3 Therapist factors associated with ERP use

Table 2 shows the characteristics of clinicians who reported frequent versus infrequent ERP use. A significantly higher proportion of clinical psychologists reported using ERP ‘often’ or ‘always’ compared to those from other professional backgrounds (93% and 52%, respectively); χ^2^ (1, *N* = 107) = 23.74, *p* < .001). Therapeutic orientation was also found to be associated with ERP use, with CBT-orientated clinicians being significantly more likely to endorse using ERP ‘often’ or ‘always’ compared to those with other therapeutic orientations (80% and 43%, respectively; χ^2^ (1, *N* = 107) = 8.69, *p* < .05). There was no significant association between ERP use and: years of experience in current role, number of OCD cases treated, or amount of supervision received per month (all *p* > .05).

**Table 2:** Characteristics of clinicians reporting frequent versus infrequent ERP use.

	Clinicians reporting frequent ERP use, *n* = 80 (%)	Clinicians reporting infrequent ERP use, *n* = 27 (%)
**Professional Background**		
Clinical Psychology (*n* = 59)	55 (93.2)	4 (6.8)
Trainee Clinical Psychology (*n* = *5)*	4 (80.0)	1 (20.0)
Nursing (*n* = 14)	6 (42.9)	8 (57.1)
Psychiatry (*n* = 9)	3 (33.3)	6 (66.7)
Social Work (*n* = 5)	3 (60.0)	2 (40.0)
Psychotherapy (*n* = 4)	2 (50.0)	2 (50.0)
Other^a^ (*n* = 11)	7 (63.6)	4 (36.4)
**Therapeutic orientation**		
CBT (*n* = 91)	74 (81.3)	17 (18.7)
Family Therapy/Systemic (*n* = 8)	1 (12.5)	7 (87.5)
Psychodynamic^b^ (*n* = 1)	1 (100.0)	–
DBT (*n* = 2)	2 (100.0)	–
Other (*n* = 5)	2 (40.0)	3 (60.0)
**Years of experience**		
<1 year (*n* = 23)	20 (87.0)	3 (13.0)
1–4 years (*n* = 28)	20 (71.4)	8 (28.6)
5–8 years (*n* = 11)	6 (54.5)	5 (45.5)
9–12 years (*n* = 22)	19 (86.4)	3 (13.6)
13–16 years (*n* = 9)	7 (77.8)	2 (22.2)
>16 years (*n* = 14)	8 (57.1)	6 (42.9)
**Number of OCD cases treated**		
1–3 (*n* = 41)	30 (73.2)	11 (26.8)
4–6 (*n* = 23)	19 (82.6)	4 (17.4)
7–9 (*n* = 10)	6 (60.0)	4 (40.0)
10–12 (*n* = 5)	5 (100.0)	–
13–15 (*n* = 2)	2 (100.0)	–
>15 (*n* = 26)	18 (69.2)	8 (30.8)
**Amount of supervision per month**		
None (*n* = 3)	2 (66.7)	1 (33.3)
<1 h (*n* = 3)	1 (33.3)	2 (66.7)
1 h (*n* = 28)	18 (64.3)	10 (35.7)
2 h (*n* = 36)	29 (80.5)	7 (19.5)
3 h (*n* = 8)	7 (87.5)	1 (12.5)
>3 h (*n* = 29)	23 (79.3)	6 (20.7)

*Note:* ERP = exposure with response prevention; CBT = cognitive behaviour therapy; DBT = Dialectical Behavioural Therapy.

a This category comprised Cognitive Behavioural Therapist (n = 1, 0.9%), Trainee CBT Therapist (n = 1, 0.9%), Educational Psychologist (n = 1, 0.9%), Family therapist (n = 1, 0.9%), IAPT Psychological Wellbeing Practitioner (n = 1, 0.9%), Occupational Therapist (n = 3, 2.7%), Substance Misuse Therapy (n = 1, 0.9%), not stated (n = 2, 1.9%).

b Inclusive of psychoanalytic psychotherapy.

Clinicians who reported using ERP ‘often’ or ‘always’ demonstrated significantly fewer negative beliefs about exposure therapy as measured by the TBES (*M* = 41.48, *SD* = 9.35), compared to those who reported low ERP use (*M* = 54.89, *SD* = 9.87; *t*(105) = 6.35, *p* < .001). Examination of TBES item-level data indicated the most commonly endorsed negative beliefs in the overall sample were ‘*Most clients have difficulty tolerating the distress exposure therapy evokes’* and *‘Arousal reduction strategies, such as relaxation or controlled breathing are often necessary for clients to tolerate the distress exposure therapy evokes’* (endorsed by 56% and 47% respectively) (see Table S3 of supplementary material)*.*

A logistic regression was performed to test the extent to which therapeutic orientation, professional background and total TBES score were unique predictors of frequent ERP use (i.e. ERP used ‘often’ or ‘always’). Together these variables explained 35% of the variance in ERP use (*R*^2^ = 34.8). Clinical psychology background was positively associated (OR = 4.40, 95% CI = 1.14, 16.98; *p* < .05) and TBES score negatively associated (OR = 0.89, 95% CI = 0.83, 0.95; *p* < .001) with frequent ERP use. Therapeutic orientation did not explain a significant proportion of the variance in ERP use (OR = 1.94, 95% CI = 0.46, 8.09; *p* > .05).

### 3.4 Use of ERP for different OCD symptoms

**Fig. 1:**
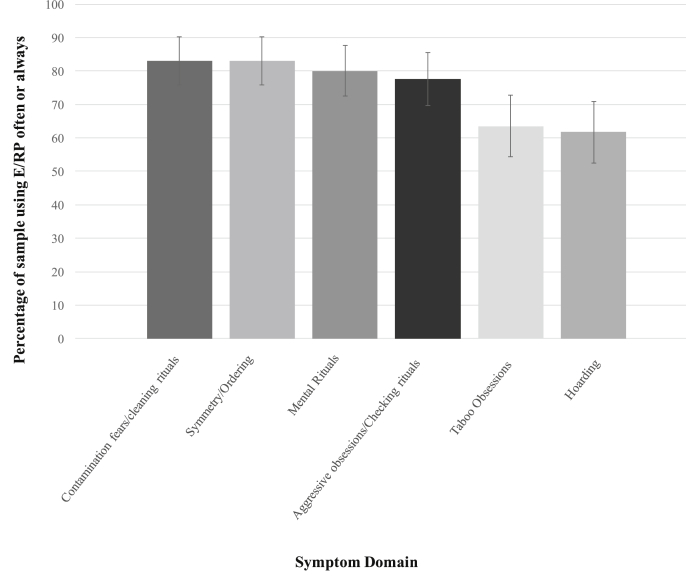
Percentage of the sample endorsing high use of exposure with response prevention for different OCD symptom domains. *Note*: Error bars represent 95% confidence intervals.

Fig. 1 shows clinicians' self-reported likelihood of using ERP for each symptom domain. Clinicians reported being most likely to use ERP ‘often’ or ‘always’ when treating contamination fears/cleaning rituals (83%, 95% CI = 75, 89), symmetry obsessions/ordering compulsions (83%, 95% CI = 75, 89) and mental rituals (80%, 95% CI = 72, 87). Compared to these three symptom domains, clinicians reported they would be less likely to use ERP to treat hoarding symptoms (62%, 95% CI = 52, 71). Clinicians were also less likely to use ERP to treat taboo obsessions (64%, 95% CI = 54, 72), compared to contamination fears/cleaning rituals and symmetry obsessions/ordering compulsions. In post-hoc exploratory analyses, we tested whether clinician characteristics predicted the likelihood of using ERP for each symptom domain in a series of logistic regressions (see Table S5). Negative beliefs about exposure (TBES score) were found to have a significant negative association with ERP use across all symptom domains. No other clinician characteristics were consistently associated with ERP use across the different OCD symptom domains.

### 3.5 Perceived barriers to ERP use

**Fig. 2:**
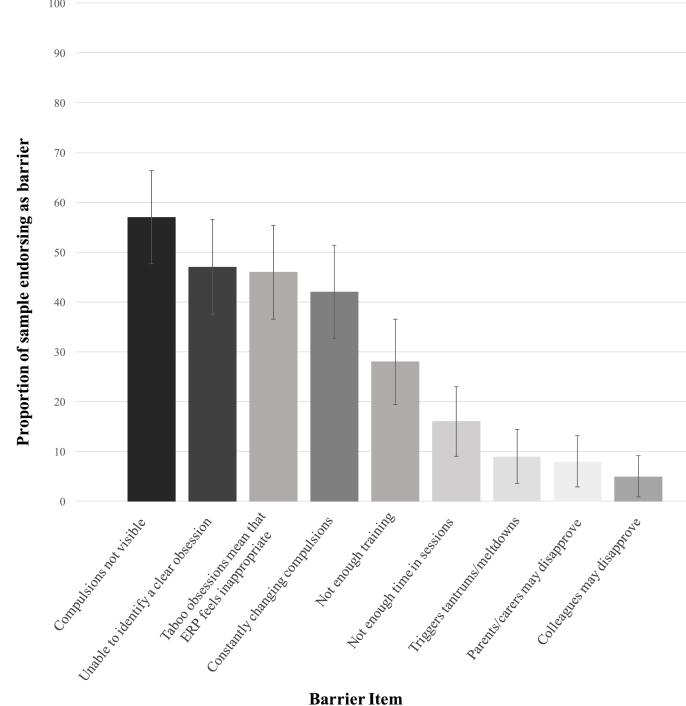
Proportion of sample endorsing specific barriers to delivering exposure with response prevention. *Note*: Error bars represent 95% confidence intervals.

Fig. 2 shows the proportion of the total sample endorsing each of the nine potential barriers to ERP use. The three most frequently endorsed barriers were compulsions not being visible (57%, 95% CI = 48, 66), the young person being unable to identify a clear obsession (47%, 95% CI = 38, 56), and the young person having taboo obsessions (46%, 95% CI = 37, 55). Of note, all four barriers that directly related to the phenomenology of OCD were endorsed more frequently than general barriers (e.g. not having enough time, 16%, 95% CI = 9, 23). In post-hoc exploratory analyses, we tested whether clinician characteristics predicted reported barriers to ERP implementation (see Table S6). These analyses showed a significant positive association between negative beliefs about exposure and almost all clinician-reported barriers, with the exception of covert compulsions. No other clinician characteristics were consistently associated with clinician-reported barriers to ERP implementation.

## 4 Discussion

The current study represents the first investigation of the use of ERP to treat paediatric OCD in the UK. In relation to our first aim, we found that 75% of clinicians reported using ERP ‘often’ or ‘always’ in their treatment of young people with OCD. This figure compares favourably with the findings from the literature regarding exposure-based treatments for anxiety disorders more generally. In addition, it is higher than the figure reported in a previous study of ERP utilisation for young people with OCD in Norway (Valderhaug et al., 2004) and is similar to the rate obtained in a German study of adult OCD therapists (Moritz et al., 2019). However, our estimate is substantially lower than that reported in a US study of adult OCD therapists (Jacobson et al., 2016). Discrepancies across studies could reflect several sample-related factors, such as characteristics of therapists (e.g. preponderance of psychologists) and the practice settings (e.g. specialist OCD/anxiety versus non-specialist).

Whilst it is promising that the majority of the overall sample reported frequently using ERP in the treatment of paediatric OCD, it is important to note that, as hypothesised, a substantial proportion of clinicians did not (almost half of non-clinical psychologists). This goes against the evidence-base and clinical guidelines, which specify ERP-based CBT as first-line treatment (NICE, 2005). Furthermore, over a third of the sample reported frequently using arousal reduction strategies when treating young people with OCD, with breathing or relaxation techniques endorsed by 41% and distraction endorsed by 36% of clinicians. This is concerning given that arousal reduction strategies are likely to undermine the efficacy of ERP by interfering with anxiety habituation (Gillihan et al., 2012).

In relation to our second aim, we found several clinician-related factors to be significantly associated with ERP use. Clinical psychologists were more likely to use ERP than participants of other professional backgrounds. This is perhaps unsurprising given that clinical psychology doctoral programmes in the UK have a key focus on CBT. It is also consistent with findings from previous research demonstrating doctoral level training is associated with a greater use of exposure-based treatments (Harned et al., 2013; Whiteside et al., 2016). As hypothesised, we also found that clinicians who hold more negative beliefs about safety, tolerability and ethicality of exposure therapy were less likely to use ERP, both in general and across all OCD symptom domains. This is in-keeping with previous studies showing such negative beliefs are associated with underuse of exposure for the treatment of anxiety disorders (Becker et al., 2004; Harned et al., 2013; Whiteside et al., 2016). In our sample, the most commonly held negative beliefs were that clients have difficulty tolerating distress evoked by exposure therapy and that arousal reduction strategies are necessary. This is consistent with our finding that over a third of clinicians reported regularly using arousal reduction strategies when treating paediatric OCD.

With respect to our third aim, clinicians reported being less likely to use ERP when treating patients with certain OCD symptom profiles. As predicted, they reported being less likely to use ERP for taboo obsessions, but also for hoarding symptoms, compared to other symptom domains. This novel finding raises the interesting question of why clinicians avoid using ERP with these symptom subtypes. It may be that clinicians assume that hoarding symptoms and taboo obsessions will be less responsive to ERP, although empirical findings suggest that this is not the case (Fernandez de la Cruz et al., 2013; Rozenman et al., 2019). For hoarding symptoms, therapists may be unsure whether they should be conceptualised as part of OCD or a distinct hoarding disorder (APA, 2013), resulting in uncertainty about treatment. For taboo obsessions, clinicians may face ethical dilemmas when designing ERP tasks, given the highly sensitive content (e.g. paedophilic obsessions). Indeed, we found that almost half of clinicians reported that they find it hard to implement ERP with taboo obsessions because ‘exposure tasks feel inappropriate’.

In relation to our final aim, as hypothesised, we found that OCD-specific factors were reported as common barriers to the successful implementation of ERP in youth with OCD. The most common barrier was covert compulsions, which was endorsed by over half of the sample. Interestingly, we also found that over 80% of clinicians reported using ERP techniques when treating mental rituals, one of the main forms of covert compulsions. Together these findings may suggest that clinicians are willing to attempt ERP when treating mental rituals, but often find it challenging to implement successfully. An alternative possibility is that clinicians find particular covert rituals (e.g. discrete tapping rituals) difficult to treat with ERP. The remaining barriers that were most commonly endorsed by clinicians were all OCD-specific (e.g. no clearly articulated obsession, constantly changing compulsions). Interestingly, the factors that Reid et al. (2017) reported as being the most frequently occurring barriers in exposure therapy for anxiety disorders in youth, including shortage of session time and concerns about parental reactions, were less commonly endorsed by the current sample. Thus, our findings suggest that the phenomenology of OCD presents unique barriers to ERP-based treatment, and clinicians may find these OCD-specific issues more problematic than the broader challenges that can be encountered with exposure therapy. Our exploratory analyses indicated that negative beliefs about exposure were associated with all clinician-reported barriers to ERP implementation, with the exception of covert compulsions. This underscores the importance of promoting a positive attitude towards exposure techniques among clinicians.

The current findings have a number of important implications for training. First and foremost, our results demonstrate a need to promote increased use of ERP, especially among those who do not have clinical psychology training. Furthermore, our findings indicate that therapist training should target unfounded negative beliefs about exposure, particularly the widely held view that OCD patients struggle to tolerate distress during ERP and that arousal reduction strategies are needed. Encouragingly, previous research has shown therapist concerns about exposure can be successfully modified during training, leading to increased implementation of exposure (Farrell, Kemp, Blakey, Meyer, & Deacon, 2016). Given our finding that clinicians often use arousal reduction strategies in the treatment of paediatric OCD, there is a clear need to educate therapists about their counterproductive effect in ERP-based treatment (Gillihan et al., 2012). Lastly, the current results show that clinicians often find specific aspects of the phenomenology of OCD challenging when delivering ERP, thereby indicating a need for specialist training in ERP for OCD, beyond broader training in exposure for anxiety. In particular, training should include use of ERP with hoarding symptoms, taboo obsessions, covert compulsions and constantly changing compulsions.

There are a number of limitations to the current study, primarily relating to the generalisability of the findings. First, although we attempted to recruit participants from a range of professional backgrounds, the majority of the sample were qualified or trainee clinical psychologists and many professions were underrepresented. Whilst this can be seen as a strength since clinical psychologists are often the main providers of CBT, it also limits the generalisability of the results to other professions. Second, given that details of the survey were distributed to prospective participants via service gatekeepers, we were unable to track the number of clinicians approached. Thus, it was not possible to calculate a response rate and estimate the impact of selection bias on our results. Third, whilst there was a range of levels of expertise within our sample, the majority of clinicians had treated a relatively small number of cases (a median of 4–6 cases). This may reflect the fact that in the UK clinicians typically work in non-specialist settings and therefore treat a wide range of disorders. Our findings may not extend to samples of more experienced or specialist clinicians. Finally, our data are based on clinician self-report. Previous studies have shown discrepancies between observed therapist behaviour and their self-report (Ward et al., 2013), and hence the current results may overestimate use of ERP. With these limitations in mind the findings should be treated as preliminary in the context of an under-researched area. Further research would be useful to see if they are replicable in larger, more varied samples.

In summary, this is the first study to demonstrate that the likelihood of clinicians using ERP to treat OCD is associated with the young person's symptom profile, with therapists being less likely to use ERP for taboo obsessions and hoarding symptoms. Furthermore, this is the first study to suggest that clinical characteristics of OCD represent key barriers to the successful implementation of ERP. Future research should test whether these issues can be successfully addressed in therapist training paradigms, in order to promote successful utilisation of ERP for paediatric OCD in routine clinical practice.

## Funding

Georgina Krebs is funded by an MRC Clinical Research Training Fellowship (MR/N001400/1).

## Statement 1: role of funding sources

Georgina Krebs is funded by a MRC Clinical Research Training Fellowship (MR/N001400/1). The MRC had no role in the study design, collection, analysis or interpretation of the data, writing the manuscript, or the decision to submit the paper for publication. According to UK research councils’ Common Principles on Data Policy, all data supporting this study will available from the authors upon request.

## Statement 2: contributors

JK contributed to survey development, data collection, data analysis, interpretation of results and writing the manuscript. AJ contributed to survey development, interpretation of results and writing the manuscript. GK contributed to conceptualization of the study, survey development, data collection, data analysis, interpretation of results and writing the manuscript.

## Statement 3: conflict of interest

The authors have declared that they have no competing or potential conflicts of interest.

## Appendix A Supplementary data

Supplementary Materials Supplementary Materials

Data Profile Data Profile
