# Supplementary material for: Clinician-reported barriers to using exposure with response prevention in the treatment of paediatric obsessive-compulsive disorder
Source: J Obsessive Compuls Relat Disord. 2020 Jan;24:100498. doi: 10.1016/j.jocrd.2019.100498 (PMC7043329; doi:10.1016/j.jocrd.2019.100498)
Supplement: Supplementary Materials [file mmc1.docx]

**SUPPLEMENTARY MATERIALS**

**Table S1.** Sample characteristics

| Variable | Proportion of total sample (%)  N = 107 |
| --- | --- |
| Professional Background |  |
| Clinical Psychology  Trainee Clinical Psychology | 59 (55.2)  5 (4.7) |
| Nursing | 14 (13.1) |
| Psychiatry | 9 (8.4) |
| Social work | 5 (4.7) |
| Psychotherapy | 4 (3.7) |
| Other* | 11 (10.3) |
| Therapeutic orientation |  |
| Cognitive Behavioural Therapy (CBT) | 91 (85.0) |
| Family Therapy / Systemic | 8 (7.5) |
| Psychodynamic inclusive of Psychoanalytic | 1 (0.9) |
| Dialectical Behavioural Therapy (DBT) | 2 (1.9) |
| None of the above | 5 (4.7) |
| Years of experience |  |
| <1 year | 23 (21.5) |
| 1 – 4 years | 28 (26.2) |
| 5 – 8 years | 11 (10.3) |
| 9 – 12 years | 22 (20.6) |
| 13 – 16 years | 9 (8.4) |
| >16 years | 14 (13.1) |
| Number of OCD cases treated |  |
| 1 – 3 | 41 (38.3) |
| 4 – 6 | 23 (21.5) |
| 7 – 9 | 10 (9.3) |
| 10 – 12 | 5 (4.7) |
| 13 – 15 | 2 (1.9) |
| >15 | 26 (24.3) |
| Amount of supervision received per month |  |
| None | 3 (2.8) |
| <1 hour | 3 (2.8) |
| 1 hour | 28 (26.2) |
| 2 hours | 36 (33.6) |
| 3 hours | 8 (7.5) |
| >3 hours | 29 (27.1) |

*Note*: Other indicates Cognitive behavioural therapist (n=1, .9%), Trainee CBT Therapist (n=1, .9%), Educational Psychologist (n=1, .9%), Family therapist (n=1, .9%), IAPT Psychological Wellbeing Practitioner (n=1, .9%), Occupational Therapist (n=3, 2.7%), Substance Misuse Therapist (n=1, .9%), not stated (n=2, 1.9%)

**Table S2. Barriers to implementing ERP when treating paediatric OCD (questionnaire items)**

| Barrier Item |
| --- |
| 1. I find it difficult to set exposure tasks because the young person cannot identify or communicate a clear obsession |
| 2. I find it difficult to set exposure tasks because the young person has taboo obsessions (e.g. sexually explicit or violent thoughts) and exposure tasks would feel inappropriate |
| 3. I find it hard to implement response prevention strategies because the young person’s compulsions are not visible (e.g. rituals) |
| 4. I do not carry out exposure with response prevention tasks because there is not enough time in sessions |
| 5. I do not carry out exposure with response prevention tasks because my colleagues might not approve of it/it involves going against workplace policies |
| 6. I do not carry out exposure with response prevention tasks because it triggers tantrums or meltdowns |
| 7. I find it difficult to implement response prevention strategies because the young person’s compulsions are constantly changing |
| 8. I do not carry out exposure with response prevention because I feel I have not had enough training to do it properly |
| 9. I do not carry out exposure with response prevention as I think that parents/carers may not approve |

**Table S3. Item level data for the Therapist Beliefs about Exposure Scale (TBES)**

| TBES Item | % endorsing TBES item  (selecting agree/agree strongly) | | |
| --- | --- | --- | --- |
|  | Clinicians reporting low ERP use  (*n* = 27) | Clinicians reporting high ERP use  (*n* = 80) | Total sample  (N = 107) |
| 1. Most clients have difficulty tolerating the distress exposure therapy evokes | 59.3 | 55.0 | 56.1 |
| 2. Exposure therapy addresses the superficial symptoms of an anxiety disorder but does not target their root cause | 55.6 | 10.0 | 21.5 |
| 3. Exposure therapy works poorly for complex cases, such as when the client has multiple diagnoses | 55.6 | 10.0 | 21.5 |
| 4. Compared to other psychotherapies, exposure therapy leads to higher dropout | 25.9 | 5.0 | 10.3 |
| 5. Conducting exposure therapy sessions outside of the office increases the risk of an unethical dual relationship with the client | 7.4 | 1.3 | 2.8 |
| 6. Exposure therapy is difficult to tailor to the needs of individual clients | 7.4 | 1.3 | 2.8 |
| 7. Compared to other psychotherapies, exposure therapy is associated with a less strong therapeutic relationship | 11.1 | - | 2.8 |
| 8. Asking the client to discuss traumatic memories in exposure therapy may retraumatise the client | 25.9 | 12.5 | 15.9 |
| 9. It is unethical for therapists to purposely evoke distress in their clients | 11.1 | 1.3 | 3.7 |
| 10. Clients are at risk of decompensating (i.e. losing mental and/or behavioural control) during highly anxiety provoking exposure therapy sessions | 33.3 | 17.5 | 21.5 |
| 11. Conducting exposure therapy sessions outside of the office endangers the client’s confidentiality | 29.6 | 6.3 | 12.1 |
| 12. Arousal reduction strategies, such as relaxation or controlled breathing, are often necessary for clients to tolerate the distress exposure therapy evokes | 77.8 | 36.3 | 46.7 |
| 13. Compared to other psychotherapies, exposure therapy places the clients at a greater risk of harm | 11.1 | - | 1.8 |
| 14. Most clients perceive exposure therapy to be unacceptably aversive | 22.2 | 1.3 | 6.5 |
| 15. Exposure therapy often causes clients’ anxiety symptoms to worsen | 18.5 | 16.3 | 16.8 |
| 16. Asking the client to discuss traumatic memories in exposure therapy may vicariously traumatize the therapist | 14.8 | 20.0 | 18.7 |
| 17. Clients may experience physical harm caused by their own anxiety (e.g. loss of consciousness) during highly anxiety-provoking exposure therapy sessions | 22.2 | 6.3 | 10.3 |
| 18. Having clients conduct exposures in their imagination is sufficient; facing feared stimuli in the real world is rarely necessary | 3.7 | 1.3 | 1.9 |
| 19. Exposure therapy is inhumane | - | - | - |
| 20. Most clients refuse to participate in exposure therapy | 18.5 | 1.3 | 5.6 |
| 21. Compared to other psychotherapies, exposure therapy increases the risk that the therapist will be sued for malpractice | - | - | - |

*Note:* ERP = exposure with response prevention

**Table S4. Reported likelihood of using ERP to treat six different OCD Symptom Domains**

| OCD Symptom Dimension | M  (*SD*) | Never  (%) | Rarely  (%) | Sometimes  (%) | Often  (%) | Always  (%) |
| --- | --- | --- | --- | --- | --- | --- |
| Contamination fears/cleaning rituals | 4.34 (1.04) | 3 | 6 | 8% | 21 | 62 |
| Symmetry/ordering | 4.27  (1.13) | 5 | 6 | 6% | 23 | 60 |
| Mental rituals | 4.15  (1.11) | 5 | 6 | 9% | 31 | 49 |
| Aggressive obsessions/checking rituals | 4.15  (1.17) | 6 | 6 | 11% | 23 | 54 |
| Taboo obsessions (e.g. inappropriate sexual thoughts) | 3.82  (1.35) | 8 | 13 | 16% | 17 | 46 |
| Hoarding | 3.71  (1.31) | 8 | 14 | 17% | 23 | 38 |

*Note:* ERP = exposure with response prevention

**Table S5: Odds ratios (95% confidence intervals) showing the association between clinician characteristics and the reported likelihood of using ERP for different OCD symptom domains**

|  | **Contamination** | **Taboo** | **Aggressive** | **Hoarding** | **Symmetry** | **Mental rituals** |
| --- | --- | --- | --- | --- | --- | --- |
| **Clinical psychologist vs other profession** | 2.18  (0.13, 36.31) | 12.48**  (1.99, 78.16) | 3.87  (0.44, 34.10) | 3.33  (0.93, 11.89) | 2.80  (0.44, 17.93) | 5.07  (0.45, 57.51) |
| **Number of years qualified** | 3.16  (0.74, 13.53) | 0.74  (0.47, 1.18) | 0.61  (0.32, 1.16) | 1.66  (0.96, 2.89) | 0.99  (0.54, 1.83) | 1.51  (0.62, 3.67) |
| **Number of OCD cases treated** | 0.75  (0.29, 1.92) | 1.45  (0.92, 2.28) | 1.82  (0.95, 3.49) | 1.03  (0.66, 1.60) | 1.08  (0.64, 1.81) | 0.92  (0.47, 1.77) |
| **Amount of supervision** | 1.23  (0.46, 3.28) | 1.09  (0.67, 1.77) | 0.58  (0.28, 1.18) | 1.32  (0.83, 2.09) | 1.13  (0.64, 1.81) | 1.91  (0.98, 3.70) |
| **CBT vs other therapeutic orientation** | 0.08  (0.00, 1.78) | 0.55  (0.12, 2.60) | 0.54***  (0.69, 4.26) | 0.25  (0.04, 1.40) | 0.43  (0.06, 3.01) | 0.06*  (0.00, 0.92) |
| **TBES** | 0.66**  (0.49, 0.88) | 0.87***  0.80, 0.94 | 0.79**  (0.69, 0.91) | 0.89**  (0.83, 0.96) | 0.87**  (0.80, 0.95) | 0.81**  (0.71, 0.93) |

Note: **p*<.05, ***p*<.01, ****p*<.001; ERP = exposure with response prevention

**Table S6: Odds ratios (95% confidence intervals) showing the association between clinician characteristics and reported barriers to using ERP when treating paediatric OCD**

|  | **Unable to identify a clear obsession** | **Taboo obsessions […]** | **Compulsions not visible** | **Not enough time in sessions** | **Colleagues may disapprove** | **Triggers tantrums / meltdowns** | **Constantly changing compulsions** | **Not enough training** | **Parents/carers may disapprove** |
| --- | --- | --- | --- | --- | --- | --- | --- | --- | --- |
| **Clinical psychologist vs other profession** | 1.57  (0.56, 4.44) | 1.92  (0.65, 5.66) | 2.37  (0.83, 6.76) | 0.48  (0.11, 2.03) | 0.65  (0.03, 12.91) | 1.26  (0.14, 11.11) | 1.52  (0.53, 4.35) | 0.53  (0.15, 1.89) | 1.53  (0.15, 15.66) |
| **Number of years qualified** | 0.62**  (0.43, 0.89) | 1.27  (0.90, 1.79) | 1.00  (0.72, 1.39) | 0.83  (0.51, 1.37) | 2.18  (0.90, 5.29) | 0.93  (0.44, 1.96) | 0.91  (0.65, 1.29) | 1.32  (0.89, 1.97) | 0.95  (0.48, 1.89) |
| **Number of OCD cases treated** | 1.11  (0.85, 1.47) | 0.80  (0.60, 1.07) | 0.78  (0.60, 1.02) | 0.99  (0.65, 1.52) | 0.71  (0.35, 1.43) | 1.31  (0.73, 2.34) | 0.77  (0.57, 1.04) | 0.77  (0.52, 1.16) | 1.50  (0.89, 2.54) |
| **Amount of supervision** | 0.74  (0.51, 1.07) | 0.91  (0.64, 1.31) | 1.14  (0.80, 1.63) | 1.03  (0.64, 1.66) | 1.33  (0.58, 3.04) | 0.72  (0.38, 1.36) | 0.95  (0.66, 1.37) | 1.08  (0.69, 1.70) | 0.62  (0.32, 1.21) |
| **CBT vs other therapeutic orientation** | 0.25  (0.05, 1.21) | 0.37  (0.09, 1.58) | 0.45  (0.11, 1.76) | 0.83  (0.17, 4.07) | 1.17  (0.07, 19.10) | 1.95  (0.20, 18.77) | 0.22*  (0.05, 0.98) | 1.27  (0.28, 5.79) | 0.59  (0.08, 4.49) |
| **TBES** | 1.06*  (1.01, 1.11) | 1.08**  (1.03, 1.14) | 1.04  (0.99, 1.09) | 1.11**  (1.04, 1.19) | 1.19*  (1.03, 1.38) | 1.21**  (1.07, 1.36) | 1.07**  (1.02, 1.12) | 1.17***  (1.09, 1.25) | 1.14*  (1.02, 1.26) |

Note: **p*<.05, ***p*<.01, ****p<.001*; ERP = exposure with response prevention.
